# Whole Blood Gene Expression Network Expression Strongly Relates to Plasma pTau217 Levels

**DOI:** 10.1002/alz70855_101170

**Published:** 2025-12-23

**Authors:** Vaibhav A Janve, Mabel Seto, Hannah M Klinger, Jane A Brown, Reisa A. Sperling, Paul S. Aisen, Robert A. Rissman, Mary Ellen I. Koran, Logan Dumitrescu, Rachel F. Buckley, Timothy J. Hohman, A4 and LEARN Study Group

**Affiliations:** ^1^ Vanderbilt Memory and Alzheimer's Center, Vanderbilt University Medical Center, Nashville, TN, USA; ^2^ Vanderbilt Genetics Institute, Vanderbilt University Medical Center, Nashville, TN, USA; ^3^ Center for Alzheimer's Research and Treatment, Department of Neurology, Brigham and Women's Hospital, Harvard Medical School, Boston, MA, USA; ^4^ Massachusetts General Hospital, Harvard Medical School, Boston, MA, USA; ^5^ Center for Alzheimer's Research and Treatment, Department of Neurology, Brigham and Women's Hospital, Boston, MA, USA; ^6^ Center for Alzheimer's Research and Treatment, Brigham and Women's Hospital, Massachusetts General Hospital, Harvard Medical School, Boston, MA, USA; ^7^ Center for Alzheimer's Research and Treatment, Brigham and Women's Hospital, Harvard Medical School, Boston, MA, USA; ^8^ Alzheimer's Therapeutic Research Institute, Keck School of Medicine, University of Southern California, San Diego, CA, USA; ^9^ Department of Radiology, Mayo Clinic, Phoenix, AZ, USA; ^10^ Vanderbilt Memory & Alzheimer's Center, Vanderbilt University Medical Center, Nashville, TN, USA; ^11^ Center for Alzheimer Research and Treatment, Brigham and Women's Hospital, Boston, MA, USA; ^12^ Melbourne School of Psychological Sciences, University of Melbourne, Melbourne, VIC, Australia; ^13^ Department of Neurology, Massachusetts General Hospital, Harvard Medical School, Boston, MA, USA; ^14^ Vanderbilt Memory and Alzheimer's Center, Vanderbilt University School of Medicine, Nashville, TN, USA

## Abstract

**Background:**

pTau217 is a well validated plasma biomarker for Alzheimer's disease (AD), sensitive to the accumulation of amyloid pathology in the preclinical stages of disease. We leveraged whole blood RNA sequencing and plasma pTau217 measures of amyloid burden to identify blood transcriptomic gene‐network changes that relate to plasma pTau217 levels in cognitively normal older adults. We then quantified the abundance of cell populations from the transcriptomic data to identify blood cell abundance alterations that correlate with plasma ptau217 levels.

**Method:**

Whole blood RNA sequencing and plasma pTau217 data were obtained from 1739 participants in the Anti‐Amyloid Treatment in Asymptomatic Alzheimer's Disease (A4) Study. RNAseq data were aligned, sorted, counted, batch normalized, and iteratively adjusted for covariates. 20,718 genes were available for analysis following quality control. Plasma samples (*n* = 1597) were analyzed for pTau217 using the Eli Lilly and Company Diagnostics Laboratory immunoassay. Samples from 724 participants (63% females, mean age 71, 61% amyloid+) with RNA sequencing and biomarker data were used for analysis. Cell fractions were quantified using CIBERSORTx (LM22 used as reference) and WGCNA was used to calculate gene co‐expression modules. Linear regression related gene module expression or cell fraction to mean pTau217 levels covarying for age, sex, education, APOE ε2 and ε4 status. Correction for multiple comparisons used the false discovery rate. Gene enrichment was performed using Gene Ontology.

**Result:**

Five gene modules were associated with plasma pTau217. For the turquoise module (β=‐0.81, p.fdr=0.005), higher expression was associated with higher *p*‐tau217 while higher expression in the blue (β=‐0.70, p.fdr=0.01), lightcyan (β=‐0.68, p.fdr=0.01), grey60 (β=‐0.64, p.fdr=0.02), and brown (β=‐0.55, p.fdr=0.04) modules were associated with lower *p*‐tau217. These modules were enriched for phagocytosis, ribonucleoprotein complex biogenesis, immunoglobulin mediated immune response, nucleosome assembly, and cytoplasmic translation pathway genes. In cell fraction analysis, higher predicted abundance of neutrophils related to ptau217 levels (β=0.33, p.fdr=0.03).

**Conclusion:**

Our results demonstrate an association between immune response and epigenetic pathway genes with plasma pTau217 levels in preclinical AD. Future work will seek to clarify whether these signatures also relate to brain amyloid PET to evaluate blood transcription networks as complementary biomarkers to improve preclinical prediction.

